# Efficient Mesoporous MgO/g-C_3_N_4_ for Heavy Metal Uptake: Modeling Process and Adsorption Mechanism

**DOI:** 10.3390/nano12223945

**Published:** 2022-11-09

**Authors:** Rasha A. AbuMousa, Lotfi Khezami, Mukhtar Ismail, Mohamed Ali Ben Aissa, Abueliz Modwi, Mohamed Bououdina

**Affiliations:** 1 Department of Mathematics and Sciences, College of Humanities and Sciences, Prince Sultan University, Riyadh 11586, Saudi Arabia; 2 College of Science, Chemistry Department, Imam Mohammad Ibn Saud Islamic University (IMSIU), P.O. Box 5701, Riyadh 11432, Saudi Arabia; 3 Department of Chemistry, College of Science and Arts, Qassim University, Ar Rass 51921, Saudi Arabia

**Keywords:** MgO/g-C_3_N_4_ sorbent, Pb^++^ and Cd^++^, pH effect, adsorption modelling

## Abstract

Removing toxic metal ions arising from contaminated wastewaters caused by industrial effluents with a cost-effective method tackles a serious concern worldwide. The adsorption process onto metal oxide and carbon-based materials offers one of the most efficient technologies adopted for metal ion removal. In this study, mesoporous MgO/g-C_3_N_4_ sorbent is fabricated by ultrasonication method for the uptake Pb (II) and Cd (II) heavy metal ions from an aqueous solution. The optimum conditions for maximum uptake: initial concentration of metal ions 250 mg g^−1^, pH = 5 and pH = 3 for Pb^++^ and Cd^++^, and a 60 mg dose of adsorbent. In less than 50 min, the equilibrium is reached with a good adsorption capacity of 114 and 90 mg g^−1^ corresponding to Pb^++^ and Cd^++^, respectively. Moreover, the adsorption isotherm models fit well with the Langmuir isotherm, while the kinetics model fitting study manifest a perfect fit with the pseudo-second order. The as fabricated mesoporous MgO/g-C_3_N_4_ sorbent exhibit excellent Pb^++^ and Cd^++^ ions uptake and can be utilized as a potential adsorbent in wastewater purification.

## 1. Introduction

Today, heavy metals, such as Lead (Pb^++^) and Cadmium (Cd^++^), exist in the natural environment and are identified as presenting a serious worldwide challenge since they cause severe impacts on public health, environment, and economy [1,2]. Both Pb^++^ and Cd^++^ are ranked among the metals that raise the most significant concerns for human health due to their persistent nature, non-biodegradability, and high toxicity even at lower exposure levels [3,4]. Furthermore, they are designated as probably carcinogens to human beings by the U.S. Environmental Protection Agency (USEPA) and the International Agency for Research on Cancer (IARC) [5]. Industrialization and sewage dumping are the main cause of the discharge of highly toxic heavy metals into natural water resources [6].

In recent years, different technologies have been utilized for wastewater purification from heavy metals, such as oxidation and reduction processes, membrane filtration, chemical precipitation, electrodialysis, biological methods, photocatalysis and adsorption, and ions exchange [7,8,9,10,11,12,13]. Of these techniques, the adsorption process has been strongly recommended in wastewater remediation due to several advantages and features, such as cost-effectiveness, simple operation, flexibility in design, and the facile regeneration of the adsorbents [14].

Among the standard materials available for the adsorption process in heavy metal uptake from contaminated water, such as activated carbon (AC), inorganic materials, modified silica gel, metal oxides, and biomaterials; the most widely used are porous metal oxides [1]. Today, metal oxide nanostructures are considered suitable adsorbents because of their inherent surface reactivity due to the presence of numerous active sites and enhanced surface area. Aside from these exceptional capabilities, the bulk form of g-C_3_N_4_ has inadequate adsorption effectiveness due to its low specific surface area [15,16], which hinders its practical use in environmental applications. Numerous techniques, such as doping [17], metal deposition [18], and heterojunctions with other semiconductors [19], have been used to overcome the disadvantages and further improve the surface properties of g-C_3_N_4_. A simple and more cost-efficient strategy is necessary for large-scale applications, even though these methods are extremely effective. In this regard, the exfoliation of g-C_3_N_4_ has shown to increase its adsorption efficacy [20]. The sonochemical method has been found to produce extraordinarily stable dispersions of g-C_3_N_4_ with high surface characteristics [21], which can then be used in a variety of cutting-edge techniques. However, metal oxides usually have a large bandgap and hence absorb only UV light, which represents 3–5% of the whole solar energy spectrum. Moreover, the purification efficiency was found to be insufficient because of the recombination probability of photoinduced electron-hole pairs [22]. Notwithstanding, researchers immobilized metal oxide nanostructures onto several substrates, including active carbon (AC) [23], carbon nanotubes [24], graphene [25], and graphitic carbon nitrate g-C_3_N_4_ [26]. As a result, the combination of metal oxides and carbon-based materials has gained significant attention in recent years since the overall efficiency was significantly enhanced for both water and air purification [1].

Graphitic carbon nitrate (g-C_3_N_4_) is a 2D carbon material with a moderate bandgap (about 2.7 eV) that recently attracted scientist’s attention with its numerous advantages, including excellent photo-degradation efficiency in visible light, high chemical and thermal stabilities, low cost, easy preparation, non-toxicity, and reliability [26,27]. Additionally, in the literature, new nanocomposites with tailored properties were developed, resulting in high photocatalytic activity, and consequently successfully used for energy production and storage, as well as the degradation of organic pollutants [22,28,29].

Magnesium oxide (MgO) nanoparticles (NPs) are ecofriendly and odorless white powders, and possess good stability under severe process conditions alongside high surface characteristics due to the evolution of edge/corner Frenkel or Schottky defects and their polyhedral nature [30]. MgO NPs exhibit interesting properties, such as high corrosion resistance, optical transmittance, low cost, and non-toxicity [31,32,33,34], and can thereby be potentially utilized in water and air purification. MgO NPs can be synthesized by several chemical and physical routes, such as co-precipitation, sol-gel, solvo-/hydro-thermal, and combustion, as well as green synthesis [35].

Herein, MgO was loaded onto 2D g-C_3_N_4_ (MGCN) to enhance the adsorption efficiency of heavy metals, specifically Pb^++^ and Cd^++^. The mesoporous MgO/g-C_3_N_4_ was fabricated by ultrasonication method and characterized using spectroscopic and analytical techniques. The adsorption capacity towards Pb^++^ and Cd^++^ ions from synthetic aqueous solution at various operational conditions, i.e., the initial concentration at different pH and contact time, was investigated.

## 2. Experimental

### 2.1. Fabrication of MGCN 

The sample was prepared by using a procedure reported before (A. Modwi et al., 2022).

### 2.2. Characterization

The particles morphology of MCGN was analyzed using scanning/transmitted electron microscopy (SEM-EDX and TEM) using Jeol S-3400 (Japan) and Tecnai G20 (USA) for TEM. X-ray diffraction (XRD) patterns were recorded to identify the crystalline phase structure by using the Bruker AXS (German) diffractometer (λ = 1.5418 Å) in the 2θ range 5–80°. The surface area and pore size of MGCN were determined by measuring N_2_ adsorption/desorption isotherms using ASAP 2020HD 88 apparatus. The surface characteristics were characterized by X-ray photoelectron spectroscopy (XPS) using VG ESCALAB 220i-XL (UK). Finally, to illustrate the adsorption mechanism, Fourier transform infrared spectroscopy (FT-IR) spectra were recorded before and after metal ions adsorption by MGCN by using Nicolet 5700 FT-IR spectrophotometer (USA). Raman spectroscopy analysis was performed using Thermo Nicolet Dispersive with a spectral resolution of 4 cm^−1^ and a spectral range of 50–500 cm^−1^.

### 2.3. Adsorption Measurements

For adsorption experiments, the 10 mg of MGCN were introduced in contact with the 25 mL Pb^++^/Cd^++^ solution at different initial concentrations (5–200 mg/L). A magnetic stirrer was employed to stirring the mixture at 400 rpm for 24 h to attain equilibrium.

For kinetics study, the experiments were conducted at fixed volume of 150 mL, initial concentration of 250 mg g^−1^, and 60 mg of MGCN. All measurements were run in the dark under magnetic stirring. At a planned timing, 5mL of the suspension was removed, centrifuged, and tested by spectrophotometer to estimate the residual concentration of Pb^++^/Cd^++^ metal ions. The Cd^++^ and Pb^++^ ions concentration was estimated using ICP Spectro Genesis Spectrometer. The adsorption equilibrium capacity ($q_{e}$ in mg g^−1^) was computed by:

(1) $$q_{e}=\frac{C_{i}-C_{e}}{m}V$$

where (*m* in g) is the adsorbate mass, (*V* in L) is the solution volume, and (*C_i_* and *C_e_* in mg L^−1^) are the initial and equilibrium concentrations of metal ions.

For the pH-dependence experiments, the metal ions concentration with an initial pH was set in solution at various pH values ranging from 1 to 8 by adding HCl (0.1 mole/L) or NaOH (0.1 mole/L).

## 3. Results and Discussion

### 3.1. MGCN Nanomaterial’s Surface Properties

**Figure 1:**
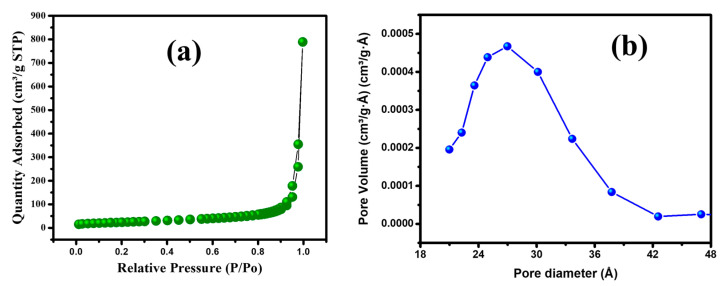
(**a**) Adsorption isotherm and (**b**) pore size distribution of MGCN sorbent.

The textural properties of the prepared MGCN, including specific surface area and pore diameter, are analyzed by nitrogen gas sorption analysis. Figure 1a depicts N_2_ adsorption–desorption isotherms. The results manifest the presence of mesopores as it displays a type IV isotherm of the IUPAC with a clear hysteresis loop (H_2_) within the range 0.0–1.0 of relative pressure. The pore size distribution is displayed Figure 1b and calculated adopting the procedure of Barret–Joyner–Halender (BJH). The as prepared MGCN exhibits 84.4 m^2^ g^−1^ surface area and 0.87 m^3^ g^−1^ average pore volume. Higher surface area and large pore volume are beneficial to improving the adsorption efficiency of metal ions from water due to the increase of the active sites at nanoparticle’ surface [26,36].

**Figure 2:**
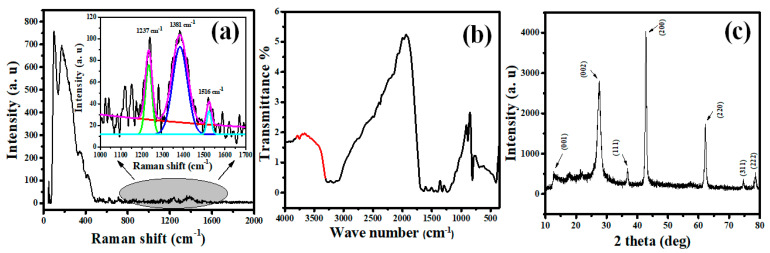
(**a**) Raman spectrum, (**b**) FTIR spectrum, and (**c**) XRD pattern of MGCN sorbent.

The Raman spectrum ranging from 0–2000 cm^−1^ is shown in Figure 2a. The peak below 400 cm^−1^ belongs to MgO [37]. The inset in Figure 2a presents three main peaks located at ~1237,  1381,  and 1516 cm^−1^ belonging to the C–N, and C=N stretching vibration modes for g-C_3_N_4_ [38,39].

The bonding and functional groups of MGCN sorbent are determined by the FTIR spectrum recorded in the range 400–4000 cm^−1^, see Figure 2b. The intense band at 810 cm^−1^ is relevant to heptazine units’ vibration, which agrees with the literature [31,40]. Moreover, the bands 1414/1486 cm^−1^ are assigned to Mg–O–Mg/Mg–O stretching, thereby confirming the formation of MgO phase. Additionally, the band at 1640 cm^−1^ is assigned to MgO. In fact, the vibrational band observed at the wavenumber at 1384 cm^−1^ corresponding to the C–O–Mg bonding, manifests the existence of an interaction between MgO and g-C_3_N_4_. This result corroborates with the previous study reported in the literature [41]. Additionally, other peaks between 3000–3500 cm^−1^ contribute to the aromatic groups’ symmetric and asymmetric vibrations of O–H and N–H bonds, implying the absorbance of water molecules [31,42]. Based on the results obtained above, the existence of g-C_3_N_4_ and the presence of its crystal structure was proven by the similarity of the distinct peaks before and after adding MgO.

The crystalline structure of the fabricated MgO/g-C_3_N_4_ sorbent is depicted in Figure 2c. The results obviously show the characteristic peaks for g-C_3_N_4_ and MgO, indicating that the loading of MgO onto g-C_3_N_4_ has been successfully achieved. For g-C_3_N_4_, the strong peak at 27.6° alongside a the small peak at 13.2◦ correspond to the (002) and (001) planes, consecutively [43]. The remaining peaks observed in the XRD pattern at 2θ = 36.8°, 42.7°, 62.12°, 74.6°, and 78.6° are indexed as (111), (200), (220), (311), and (222) reflections, belonging to the cubic structure of MgO phase in agreement with JCPDS card No. 78–0430 [31], which is consistent with Raman and FTIR analyses. The Scherrer formula (D = Kλ/βcosθ where β is the full-width at full maximum) has been utilized to estimate the mean crystallite size (D) of MGCN sorbent using the main peak (002) of g-C_3_N_4_ and (200) of MgO. The calculated crystallite size is found to be 7.83 nm g-C_3_N_4_ and 23.91 nm for MgO, which corroborates with previous studies [26]. The XRD pattern does not reveal any additional peaks, indicating the absence of impurities and confirms the high purity of the prepared MGCN sorbent.

**Figure 3:**
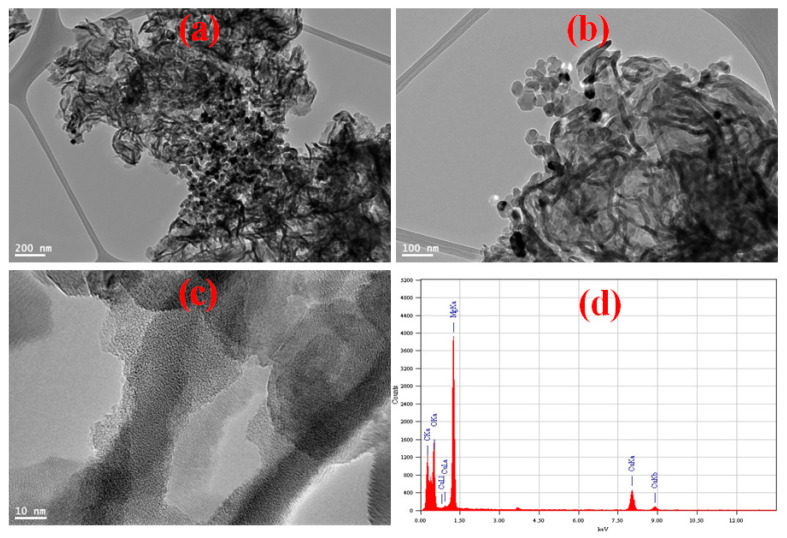
(**a**–**c**) TEM micrographs, (**d**) EDX spectrum with the corresponding chemical composition of MGCN sorbent.

TEM images of MGCN shows 2D irregular sheet or flakes-like morphology corrugated around 30 nm of thickness, as depicted in Figure 3a–c. The MgO particle size in MGCN nanocomposite is around 25–50 nm, in agreement with the crystallite size estimated by XRD analysis. Moreover, it can be observed that MgO nanoparticles are evenly and highly dispersed onto sheet-like g-C_3_N_4_, which form self-active sides through the MGCN surface. Figure 3d presents the elemental composition as obtained by EDX analysis. The EDX spectrum reveals the absence of any impurities other than C, N, O, and Mg.

**Figure 4:**
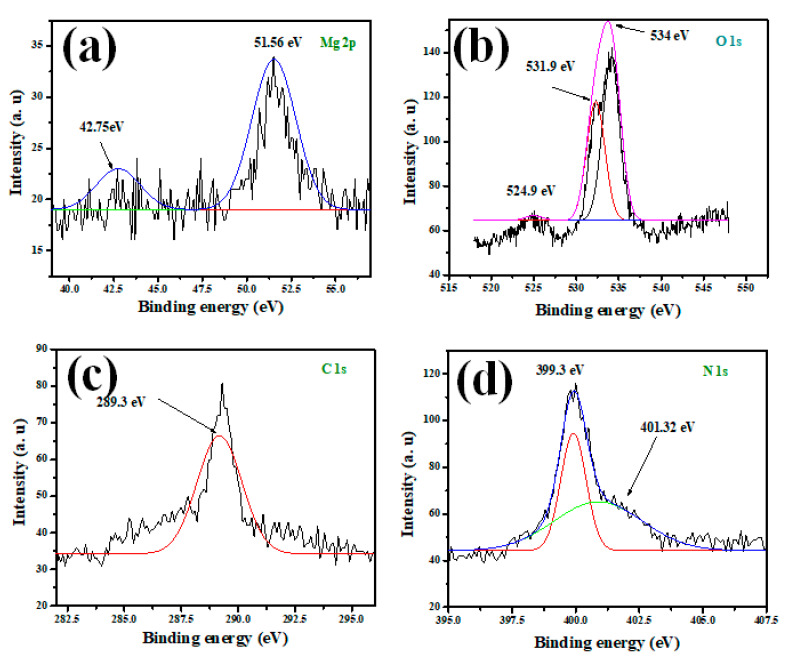
XPS spectra of (**a**) Mg-2p, (**b**) O-1s, (**c**) C-1s, and (**d**) N-1s for MGCN sorbent.

High-resolution X-ray photoelectron spectroscopy (HR-XPS) has been utilized to evaluate the chemical constituents of the mesoporous MGCN nanocomposite. The individual peaks of Mg, O, C, and N elements are depicted in Figure 4 to confirm the physical binding of MgO onto the g-C_3_N_4_ surface. The peak at 289.3 eV is assigned to C-1s, as illustrated in Figure 4c, and corresponds to the sp^2^ hybridized C = N species covalent bond (A. Modwi et al., 2022; Toghan and Modwi, 2021). The peak at 51.56 eV, suggesting the characteristic Mg-2p peak for MgO (Figure 4a); likewise, the signals of O-1s at 534 eV and O-2s at 42.75 eV are attributed to Mg-O bonding. Furthermore, the two signals N-1s observed in Figure 4d at 399.3 eV and 401.32 eV are ascribed to C–N–C of sp^2^ hybridized nitrogen and N–(C)_3_ tertiary nitrogen, respectively. The O-1s depicted in Figure 4b reveals a supplementary signal at 531.98 eV, corresponding to C-O bonding. The XPS analysis of the prepared MGCN nanocomposite also confirms its high purity, since only the constituents of the composite C, N, Mg, and O are detected, which corroborates with the results reported in the literature [40,43].

### 3.2. MGCN Adsorption Study

#### 3.2.1. Comparative Analysis of the Adsorption Capabilities of MgO, g-C_3_N_4_, and MGCN

**Figure 5:**
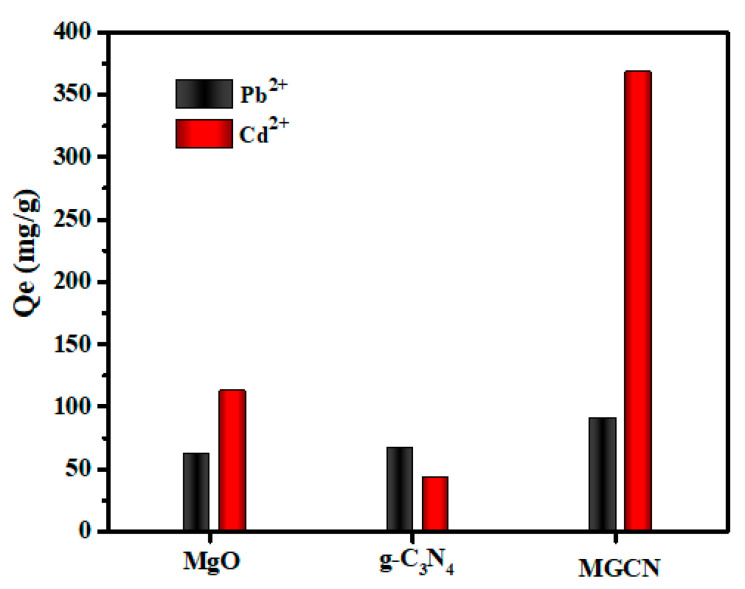
Pb^++^ and Cd^++^ adsorption capacities of MGCN, g-C_3_N_4_, and MgO with an initial Pb^++^ and Cd^++^ concentration of 45 ppm and initial pH = 5.

To compare the Pb^++^ and Cd^++^ adsorption capacities of MgO, g-C_3_N_4_, and MGCN, a series of adsorption experiments were conducted at a fixed initial heavy metals concentration of 45 mg g^−^^1^ and a pH value 5, and the obtained results are given in Figure 5. It is interesting to note that MGCN demonstrates selective adsorption towards Cd^++^ compared to Pb^++^, i.e., the adsorbed amount is more than three times higher (90.83 mg g^−^^1^ for Pb^++^ and 367.73 mg g^−^^1^ for Cd^++^). Furthermore, the adsorption capacity of MGCN towards Cd^++^ is substantially higher than the respective capacities of pure g-C_3_N_4_ (42.50 mg g^−^^1^) and MgO (112.43 mg g^−^^1^). For Pb^++^, the adsorption capacity increases slightly, reaching 62.13 mg g^−^^1^ for MgO followed by 67.13 mg g^−^^1^ for g-C_3_N_4_ then up to 90.83 mg g^−^^1^ for MGCN. The obtained results highlight the synergistic effect as consequence of combining of 3D MgO NPs with 2D g-C_3_N_4_ nanosheets.

#### 3.2.2. Impact of pH on the Uptake Process

**Figure 6:**
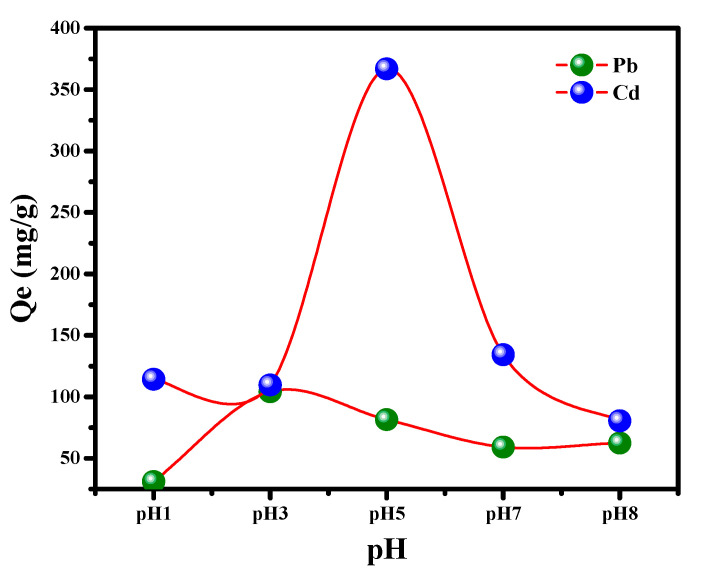
Effect of pH on the removal of Pb^++^/Cd^++^ by MGCN nanosorbent.

The pH is a well-known factor affecting the heavy metal uptake from an aqueous medium because it influences the charge transfer onto solid/liquid interface. The pH effect on Pb^++^/Cd^++^ ions uptake has been investigated within the range 1.0–9.0. It can clearly be observed from Figure 6 that Pb^++^/Cd^++^ adsorption onto MGCN sorbent is pH-dependent. There is a noticeable increase in the adsorption of Pb^++^ and Cd^++^ uptake as the solution pH increases to reach its maximum capacity at pH 5 and 3, respectively, then followed by a gradual reduction in the adsorption for greater pH values. The reduction in the adsorption rate at lower pH elucidates the competitive adsorption between H+ available in the solution and cations Pb^++^/Cd^++^ [41,44,45]. The solubility of metal ions like Pb^++^ and Cd^++^ varies with the pH value of the solution. At lower pH values, Pb^++^ and Cd^++^ are extensively soluble as Pb^++^ and Cd^++^ free ions, as well as Cd(OH)^+^ and Pb(OH)^+^ [46]. Additionally, at higher pH values, the cations of Pb^++^ and Cd^++^ precipitate as metal hydroxides Pb(OH)^2^ and Cd(OH), respectively [47]. At higher pH, a reduction of the adsorption is noted, corresponding to the formation of metal hydroxides [48]. Therefore, pH 3 and 5 are chosen as the optimum pH conditions for the consequent experiments’ Pb^++^ and Cd^++^ uptake.

#### 3.2.3. Adsorption Kinetics

**Figure 7:**
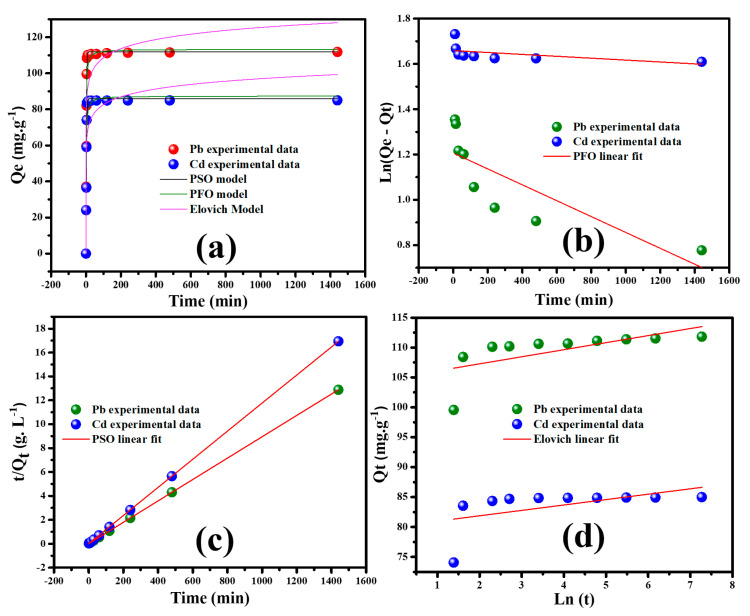
(**a**) Effect of contact time, linear fit of (**b**) pseudo–first order, (**c**) pseudo–second order, and (**d**) Elovich on the sorption of Pb^++^/Cd^++^ onto MGCN sorbent.

The study of kinetics is essential to better elucidate the adsorption process. The adsorption rate is crucial in the metal ions’ adsorption onto the adsorbent. Hence, the time-dependent adsorption experiment has been carried out to estimate the adsorption rate, and the obtained results are illustrated in Figure 7a. A contact time ranging from 0 min to 24 h is adopted during the adsorption experiments. It is clearly observed that the adsorption capacity increases sharply with the contact time, and in less than 50 min, the equilibrium is attained.

The presence of active metal sites and facile accessibility without obstacles mainly affects the sorbent’s kinetic characteristic. In other words, the adsorption mechanism depends on the mass transfer process and the chemical reaction [6]. Therefore, Lagergren pseudo-first/-second order and Elovich are the three general models employed for the adsorption kinetics of Pb^++^/Cd^++^ ions from water by MGCN nanocomposite, as shown in Figure 7b–d. The models’ kinetic linear equations are listed in Table 1 while the obtained parameters are given in Table 2. The fit of the experimental data with the theoretical models is validated by expressing the correlation coefficient *r*^2^, i.e., the higher *r*^2^ value, the more suitable the model for Pb^++^/Cd^++^ uptake onto MGCN sorbent.

Figure 7b illustrates the kinetic plots of the pseudo-first order model in the form of $\ln\;\left(Q_{e}\;–\;Q_{t}\right)$ vs. t. This linear plot is used to determine the first order rate constant $k_{1}$ and $q_{e}$, corresponding to the slope and intercept, respectively. The failure of the first order model to describe the adsorption process is evidenced from the obtained remarkably poor correlation coefficient $r^{2}$ values: 0.7880 and 0.8649 for Pb^++^ and Cd^++^ ions, respectively.

The kinetic graphs of the pseudo-second order model are shown in Figure 6c. From the plots $t/Q_{t}$ vs. t, straight lines are obtained for both Pb^++^ and Cd^++^ with a high r^2^ value of 0.999 is found for both Pb^++^/Cd^++^. Subsequently, the values of *k*_2_ and $q_{e}\;$ from the slope and the intercept, respectively, are evaluated. These obtained results, as given in Table 2, indicate that the adsorption of Pb^++^/Cd^++^ ions onto MGCN sorbent is better suited to the pseudo-second order kinetics rather than pseudo-first order kinetics.

Furthermore, the Elovich model has been also tested by plotting *q_t_* versus Ln *t*, as illustrated in Figure 7d. The correlation coefficient r^2^ = 0.9864 for Pb^++^ uptake onto MGCN sorbent is like the value acquired by the pseudo-second order model and more significant than the value taken from the pseudo-first order model. 

The obtained results, as shown in Figure 7a–d and Table 2, indicate that the chemisorption mainly controls the adsorption process based on valence force by electron participating between heavy metal ions and the adsorbents. On the contrary, the low value of r^2^ = 0.6744 obtained for Cd^++^ uptake onto MGCN sorbent signifies the bad fit of the adsorption process.

#### 3.2.4. Intra-Particle Diffusion Study

**Figure 8:**
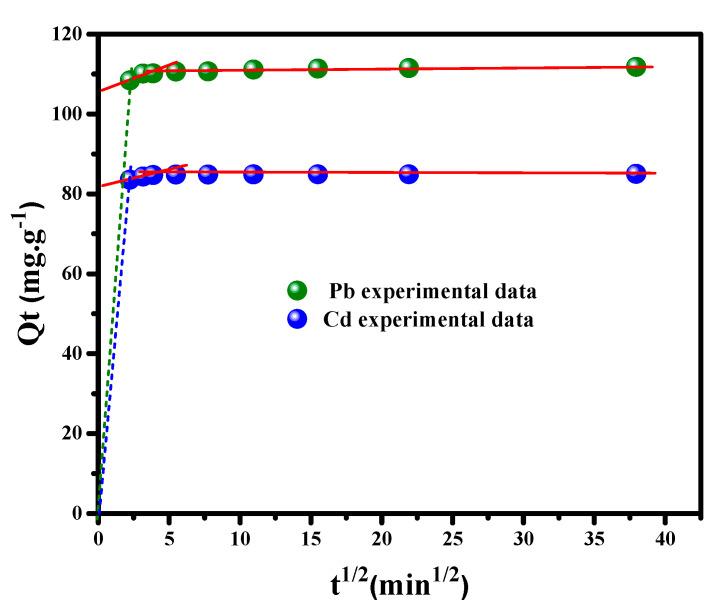
Intra-particle-diffusion (*q_t_* vs. *t*^1/2^) for Pb^++^/Cd^++^ removal by MGCN sorbent.

Weber and Morris’s diffusion theory has been examined to investigate the intra-particle diffusion model, defined as a restrictive step for some adsorption processes. Based on this theory (equation listed in Table 1), the kinetic parameters and fitting parameters can be determined from the plot of $Q_{t}$ vs. t12 (Figure 8), where the intra-particle diffusion constants $k_{dif}$ and C acquired from the plot’s slope and intercept, respectively, are presented in Table 2. It is apparent from the linearity of plots that Pb^++^ and Cd^++^ ions uptake onto the MGCN surface exhibit a high efficiency. The intra-particle diffusion model is an important mode of diffusion due to the superior values of the correlation coefficient, i.e., *r*^2^ = 0.9941 and 0.9818 for Pb^++^ and Cd^++^, respectively. The *C* factor is the parameter indicating the boundary layer diameter, and its higher values demonstrate the effect of the solution boundary layer on Pb^++^/Cd^++^ uptake. A substantial value of *C* in the second step is obtained compared to the first step, which manifests the occurrence of heavy metal ion uptake by MGCN through the intraparticle diffusion phenomenon [26].

#### 3.2.5. Adsorption Isotherms

**Figure 9:**
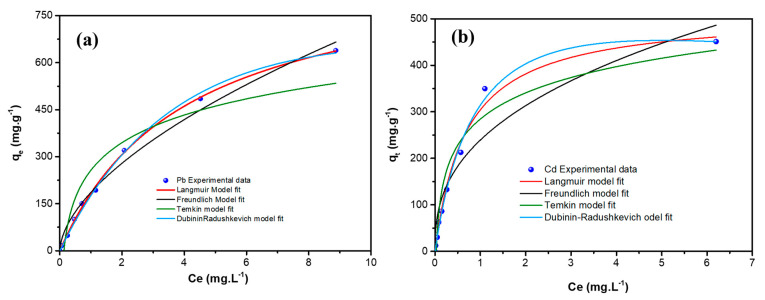
Non-linear Adsorption equilibrium isotherms of (**a**) Pb^++^ and (**b**) Cd^++^ metal ions onto MGCN sorbent.

The adsorption isotherms describe the equilibrium relationship between the heavy metal ions and the sorbent under given conditions. Figure 9 shows the adsorption isotherms of Pb^++^/Cd^++^ ions adsorption by MGCN.

**Figure 10:**
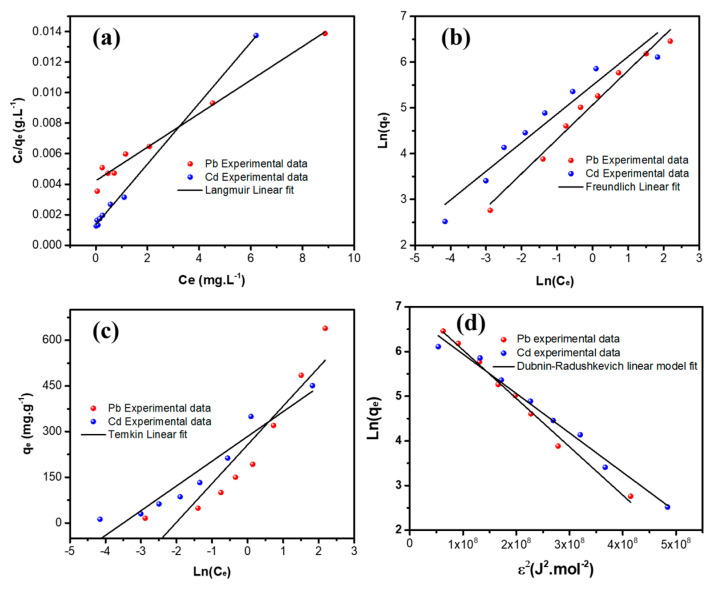
Linear adsorption equilibrium isotherms of Pb^++^/Cd^++^ onto MGCN sorbent fitted with the (**a**) Langmuir, (**b**) Freundlich, (**c**) Temkin, and (**d**) Dubinin–Radushkevich models.

The Langmuir, Freundlich, Temkin, and Dubinin–Radushkevich models are the four different isotherms’ models used to fit the obtained experimental data, as illustrated in Figure 9 and Figure 10. Both linear and non-linear equations of the isotherm are listed in Table 3, and the obtained parameters with the corresponding regression coefficient *r*^2^ are given in Table 4 for both metal ions. Obviously, the Langmuir adsorption isotherm model manifests the highest regression coefficient, i.e., *r*^2^ values are 0.9989 and 0.9909, for the adsorption of Pb^++^ and Cd^++^ onto MGCN. More it is a favorable adsorption process since the RL value is positive for both metal ions (Table 4). In contrast, Freundlich and Temkin’s models are found to be unsatisfactory, and the values of *r*^2^ are below 0.99. These findings demonstrate that the Langmuir adsorption isotherms model is a well-fitting model.

Moreover, it should be pointed out that the D-R equilibrium model can also be regarded as suitable to describe the experimental adsorption data of Pb^++^ and Cd^++^ by MGCN, considering the same criterion, i.e., *R*^2^ = 0.9924 and 0.9852, for Pb and Cd metal ions, respectively (Table 4). Depending on the D-R isothermal model’s energy value (E), the adsorption process is generally communed to physical or chemical [51]. Physisorption ensures whether the E value is less than 8 kJ.mol^−1^ [52]. In contrast, chemisorption occurs for energy values ranging from 8 to 16 kJ.mol^−1^ [52]. The obtained D-R isothermal model’s energy values are 6.795 and 7.525 kJ.mol^−1^ for Pb^++^ and Cd^++^ removal, respectively, manifesting that the metal ion adsorption mechanism onto MGCN occurs by physisorption.

**Table 3:** Equilibrium models.

Equilibrium Model	Linear Form	Non-Linear Form
Langmuir [53]	$\frac{C_{e}}{q_{e}}=\frac{1}{q_{m}K_{L}}+\frac{C_{e}}{q_{m}}$	$q_{e}=\frac{q_{max}K_{L\;}C_{e}}{1+K_{L\;}C_{e}}$
Freundlich [54]	$lnq_{e}=lnK_{F}+\frac{1}{n}lnC_{e}$	$\;\;q_{e}$ *=* $k_{F}C_{e}^{1/2}$
Temkin [55]	$q_{e}=\frac{RT}{B}lnK_{T}+\frac{RT}{B}lnC_{e}$	$q_{e}=\frac{RT}{B}ln(K_{T}C_{e})$
Dubnin-Radushkevich [53]	$lnq_{e}=lnq_{m}-K\varepsilon^{2}$ $\varepsilon =RTLn\left(1+\frac{1}{C_{e}}\right)$, $E=\frac{1}{\sqrt{2B}}$	$q_{e}=q_{m}e^{-K\varepsilon^{2}}$

**Table 4:** Isotherm parameters for the adsorption of Pb^++^/Cd^++^ ions onto MGCN sorbent.

**Equilibrium Model**	**Parameters**	**Pb^++^**	**Cd^++^**
Langmuir	*q_m_* (mg g^−1^)	927.81	511.55
	*K_L_* (mg g^−1^)	0.247	1.467
	*R_L_* (L mg^−1^)	0.0043	0.0013
	*r* ^2^	0.9989	0.9909
Freundlich	n	1.71	2.57
	*K_F_* (L mg^−1^)	186.34	239.24
	*R* ^2^	0.9867	0.9168
Temkin	*B* (J mol^−1^)	19.51	30.51
	*K_T_* (L mg^−1^)	7.55	33.14
	*r* ^2^	0.8879	0.9367
Dubnin–Radishkevich	*q_m_* (mg g^−1^)	1234	922
	*K* (mol kJ^−1^)^2^	1.08 × 10^−8^	8.83 × 10^−9^
	*E* (kJ mol^−1^)	6.795	7.525
	*r* ^2^	0.9924	0.9852

Khezami et al. [56,57] proved by studying the Cd (II) ions adsorption onto pure and Al-doped ZnO nanoparticles, is a spontaneous physisorption process. Similarly, Mustapha et al. [58] confirmed the physisorption nature of the Pb^2+^ adsorption onto a RuO_2_–ZnO nanocomposite.

### 3.3. Adsorption Mechanism

**Figure 11:**
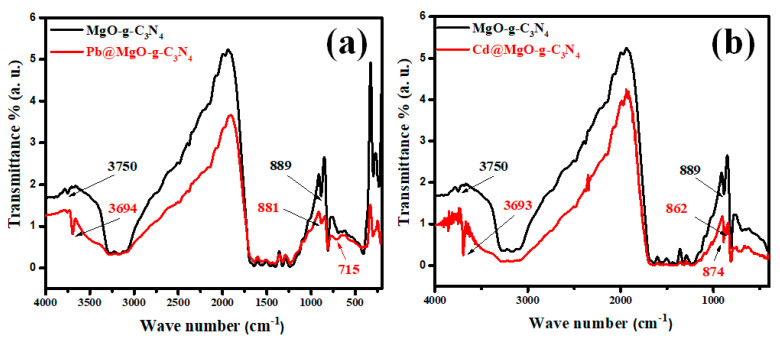
FTIR spectra of MGCN sorbent before and after adsorption of (**a**) Pb^++^ and (**b**) Cd^++^.

The adsorption mechanism of Pb and Cd metal ions onto the MGCN surface is clarified by recording the FTIR spectra of MGCN before and after the adsorption process, as depicted in Figure 11. The spectra depict different changes and shifts in the peaks. A shift in the peak at 3750 cm^−1^ to 3697 cm^−1^ after Pb^++^ adsorption and into 3693 cm^−1^ after Cd^++^ adsorption, signifying that (O–H) contributes to the adsorption of metal ions [59]. The displacement of the OH stretching vibration band towards a higher/lower wavenumber confirms the interaction of Pb^++^ and Cd^++^ ions with oxygen atoms present at the surface of MGCN nanosorbent through surface complexation [60,61]. Moreover, the apparition of the (Pb–O) vibration band at 715 cm^−1^ and (Cd–O) stretching vibration at 862 cm^−1^ in the FTIR spectra of MGCN after adsorption [62,63], confirms the interaction of Pb^++^ and Cd^++^ ions with the oxygen atoms of MgO. Additionally, a broadened and shift after adsorption is noticeable within the range 3000–3500 cm^−1^ to a higher wavenumber, indicating that amino group stretching bands are involved during the adsorption through cation-bonding ([61,64].

**Figure 12:**
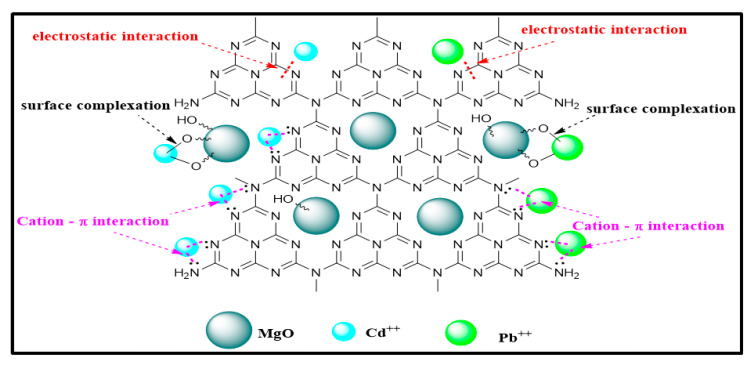
The main sorption mechanism of Pb++ and Cd++ ions onto MGCN sorbent.

Moreover, the peak at 889 cm^−1^ ascribed triazine ring (C_3_N_3_) mode also slightly shifted to 881 cm^−1^ for Pb^++^ and 874 cm^−1^ for Cd^++^. The π  delocalized electron of the tri-s-triazine ring (C_3_N_3_) acts as Lewis’s base, as mentioned by Xiao et al., while the metal ions act as Lewis’s acid [41]. Accordingly, the electrostatic interaction involved during adsorption of heavy metal ions onto MGCN occurs through the Lewis base–acid interaction. Figure 12 illustrates the main sorption mechanism of Pb++ and Cd++ ions onto the MGCN sorbent.

The adsorption optimum experimental conditions for removing Pb and Cd metal ions through adsorption onto MGCN alongside other previously investigated nanocomposites are listed in Table 5. The MGCN prepared by sonification demonstrates excellent potential for treating wastewater from Pb^++^ and Cd^++^ compared to other sorbents reported in the literature. Although the decreased size of MgO has a high surface area, it unavoidably reduces the stability. According to van der Waals force/other interactions, the aggregation of particles with a small diameter is likely to occur, consequently causing a decrease in MgO adsorption performance with smaller diameters/large surface area [65]. Furthermore, the formation aggregates result in the reduction of active sites, and hence a decline in the adsorption rate. In 2021, Fouda et al. investigated the adsorption of several heavy metal ions onto MgO-NPs and the results showed a moderate removal percentage for Cd and Pb metal ions around 74.1% ± 1.8% and 72.7% ± 1.3%, respectively. Nonetheless, although the achieved efficiency is relatively good, the adsorption reached equilibrium after a longer contact time of around 180 min using a 100 mg dosage of MgO-NPs, which are considered unsatisfactory optimal adsorption conditions [66]. While in this study, the as fabricated composite MGCN showed a better adsorption performance toward Cd and Pb metal ions with a removal efficiency around 97% and 95.6%, respectively, at 60 mg of MGCN within less than 50 min. This is attributed to the improvement in the number of active sites with much lower tendency to aggregation of MGCN nanostructured composite. Therefore, mesopores MGCN can be considered as a promising adsorbent in wastewater purification. The present MGCN nanostructured composite with high surface area and large pore size shown as a promising sorbent to eliminate other heavy metal ions from aqueous solutions.

## 4. Conclusions

The fabricated mesoporous MGCN using the ultrasonication method are utilized for Pb^++^ and Cd^++^ metal ion removal from aqueous solutions. The adsorption of both metal ions onto MGCN sorbent is affected by the operating factors, namely solution pH, initial metal ion concentration, and sorbent dose. The results demonstrated that the MGCN sorbent has a high surface area 84.4 m^2^/g and pore volume of 1.22 cm^3^/g, fast adsorption equilibrium for Pb^++^/Cd^++^ in less than 50 min, and a high adsorption capacity of 114 and 90 mg g^−1^ for Pb^++^ and Cd^++^, respectively. Moreover, the adsorption isotherm/kinetics models perfectly fit with the Langmuir isotherm/pseudo-second order models. A plausible mechanism of Pb^++^/Cd^++^ adsorption onto mesoporous MGCN is elucidated. The Pb^++^/Cd^++^ adsorption mechanism was associated with surface complexation, cation-bonding, and electrostatic interaction, as indicated by FTIR analysis. The mesoporous MGCN sorbent exhibits excellent Pb^++^/Cd^++^ uptake and can be potentially utilized in wastewater purification. 

## Figures and Tables

**Table 1:** Kinetics models for the adsorption of Pb^++^/Cd^++^ by MGCN sorbent.

Kinetics Model	Kinetic Equation
Pseudo-first order [49]	$\ln\;(q_{e}-q_{t})=\ln q_{e}-k_{1}t$
Pseudo-second order [50]	$\frac{t}{q_{t}}=\left[\frac{1}{k_{2}q_{e}^{2}}\right]+\frac{1}{q_{e}}t$
Elovich [50]	$q_{t}=\frac{1}{\beta }\ln\left(\alpha \beta \right)+\frac{1}{\beta }\ln t$
Intra-particle Diffusion [50]	$q_{t}=k_{dif}t^{1/2}+C$

**Table 2:** Kinetic models parameters for the adsorption of Pb and Cd by MGCN sorbent.

Pseudo-Second Order Model						
Metal ion	*q_e_*_(Exp)_^a^ (mg g^−1^)	*t*_1/2_ (min)	*h*_0_ (mg g^−1^ min^−1^)	*q_e_*_(Cal)_^b^ (mg g^−1^)	*K*_2_ × 10^2^ (g mg^−1^ min^−1^)	*r* ^2^
Pb^++^	114	0.84	133.34	112.4	1.06	0.9999
Cd^++^	90	0.75	112.36	84.8	1.56	0.9999
Pseudo-First Order model				Elovich’s model		
Metal ion	*q_e_*(Cal) ^b^ (mg g^−1^)	*K*_1_ (min^−1^)	*r* ^2^	*k_b_* (L g^−1^)	*α* × 10^2^	*r* ^2^
Pb^++^	3.3	1 × 10^−^^3^	0.7880	1.252	5.8	0.9864
Cd^++^	5.1	2 × 10^−^^5^	0.8649	1.245	1.0	0.6744
Intra-particle diffusion/transport model						
Metal ion	*k*_dif1_ (mg g^−1^ min^−1/2^)	C1	*r* ^2^	*k*_dif2_ (mg g^−1^ min^−1/2^)	C2	*r* ^2^
Pb^++^	59.92	23.13	0.9941	0.225	109.4	0.9731
Cd^++^	50.80	29.7	0.9818	0.005	84.83	0.9527

^a^: Experimental data; ^b^: Calculated data from models.

**Table 5:** Adsorption characteristics of MGCN and other nanosorbents for the removal of Pb^++^ and Cd^++^.

Adsorbents	Surface Area (m^2^/g)	q_e_ (mg g^−1^)	Removal Efficiency (%)	Optimum pH and Initial Concentration Ci	Reference
g-C_3_N_4_	111.2	Cd: 123.205	80%	Not mentioned Ci = 20 mg L^−1^	[41]
MgO	Not mentioned	Cd: 135	74.1%	Not mentioned Ci = Not mentioned 100 mg dosage	[66]
Inorganic nanocomposites with different iron concentration	649–680	Cd: 1.12	90–92%	6.5 Ci = 30 mg/L	[67]
Modified orange peel	Not mentioned	Cd: 13.7	85%	5 Ci = 20 mg L^−1^	[6]
NiFe-CO_3_-LDH-NGO composite	151	Cd: 971	>95%	5 Ci = 10–1000 mg L^−1^	[14]
Natural kaolinite clay	3.7	Cd: Not mentioned	94%	7 Ci = 20 mg L^−1^	[4]
MGCN	84.37	Cd: 511.55	97%	5 Ci = 5–200 mg L^−1^	This paper
Fe_2_O_3_/TiO_2_	130	Pb: Not mentioned	94%	6.5 Ci = 35 mg L^−1^	[68]
CSt-ZnO nanocomposite	185.21	Pb: 256.4	68%	6 Ci = 19.97 mg L^−1^	[69]
g-C_3_N_4_	111.2	Pb: 136.571	80%	Not mentioned Ci = 20 mg L^−1^	[41]
MgO	Not mentioned	Pb: 148.6	72.7%	Not mentioned Ci = not mentioned 100 mg dosage	[66]
Silica-Coated Magnetic Nanocomposites	271.0 m^2^	Pb: 14.9	Not mentioned	4–6 Ci = 2–120 mg L^−1^	[70]
Modified orange peel	Not mentioned	Pb: 73.53	96%	5 Ci = 20 mg L^−1^	[6]
Fe_3_O_4_@SiO_2_-EDTA	24.07	Pb: 125.24	Not mentioned	5.3 ± 0.1 Ci = 100 mg L^−1^	[71]
MGCN	84.37	Pb: 927.81	95.6%	3 Ci = 5–200 mg L^−1^	This paper

## Data Availability

Not applicable.
